# Dual-Polysaccharide Reinforced Pickering Emulsion Gels for Tailoring Microstructure and Enhancing 3D Printing Performance

**DOI:** 10.3390/foods15142482

**Published:** 2026-07-13

**Authors:** Haoyu Zhou, Xingui Song, Zefan Zhang, Henghao Li, Wei Yang

**Affiliations:** 1 School of Food Science, Henan Institute of Science and Technology, Xinxiang 453003, China; 2 School of Food Science and Biotechnology, Zhejiang Gongshang University, Hangzhou 310018, China

**Keywords:** high internal phase Pickering emulsion, whey protein isolate, self-assembled particles, κ-carrageenan, curdlan gum, 3D printing

## Abstract

Three-dimensional (3D) food printing enables personalized fabrication but requires materials with suitable printing properties. This study developed high internal phase Pickering emulsions (HIPPEs) stabilized by co-assembled whey protein isolate (WPI) with κ-carrageenan (κ-CA) in a binary system and with both κ-CA and curdlan gum (CG) in a ternary system. The aim was to clarify the distinct roles of anionic and neutral polysaccharides in regulating emulsion printability. Incorporation of 1.2% κ-CA and 1.6% CG optimized the three-phase contact angles of the binary and ternary particles to 78.84 ± 0.87° and 86.22 ± 0.74°, respectively. The ternary system exhibited significantly greater oil-phase wettability (*p* < 0.05). Rheological and textural analyses showed that κ-CA concentration primarily governed yield stress and self-supporting capacity in the ternary system, with an optimum at 1.2%. In contrast, CG incorporation was essential for improving thixotropic recovery and printing accuracy, with an optimum at 1.6%. The optimized ternary HIPPEs exhibited excellent 3D printing accuracy with a food-grade oil phase and surpassed the binary system in structural integrity and shape fidelity. These findings clarify the distinct yet complementary roles of anionic and neutral polysaccharides in modulating HIPPEs printability and provide a rational material-design strategy for developing high-performance food 3D-printing system.

## 1. Introduction

Three-dimensional printing has emerged as a transformative digital manufacturing technology in the food industry, enabling on-demand customization of complex structures for individual nutritional needs, aesthetic preferences, and swallowing disorders [1]. The printability of food inks largely depends on their rheological properties, which must provide balanced extrudability during deposition, high shape fidelity immediately after printing, and long-term structural stability [2,3,4,5].

HIPPEs have attracted considerable interest as promising ink matrices because of their tunable rheology and high stability [6,7,8]. Among various stabilizers, food proteins such as whey protein isolate (WPI) are widely used to prepare edible HIPPEs because of their excellent biocompatibility, surface activity, and nutritional value [9]. However, pristine protein-stabilized HIPPEs generally exhibit poor thermal stability and low gel strength, resulting in inadequate shape retention after printing and limiting their application in 3D food printing.

Polysaccharides are widely used to improve protein-stabilized emulsions through their thickening, gelling, and interfacial regulatory functions [6,7] (Li et al., 2024; Wei et al., 2024). Among them, κ-carrageenan (κ-CA), an anionic sulfated polysaccharide, forms electrostatic complexes with proteins under acidic conditions, thereby enhancing interfacial adsorption and emulsion stability [10] (Du & Meng, 2024). Compared with other anionic polysaccharide–protein systems, ĸ-carrageenan–protein stabilized Pickering emulsions exhibit superior printing accuracy during 3D printing [7,8]. However, carrageenan undergoes viscosity reduction and gel-to-sol transition at high temperatures, compromising emulsion stability. In contrast, curdlan gum (CG), a neutral polysaccharide, forms heat-induced gels that suppress emulsion droplet coalescence and collision at elevated temperatures. Recent studies have also shown that CG possesses intrinsic emulsifying activity and acts synergistically with proteins to stabilize emulsions [11,12,13].

Despite these advances, most studies have focused on modifying HIPPEs using a single polysaccharide. The synergistic effects of polysaccharide combinations remain poorly understood, particularly the complementary roles of anionic and neutral polysaccharides in regulating interfacial assembly, rheological behavior, and 3D-printing performance. Moreover, systematic comparisons between binary (protein + anionic polysaccharide) and ternary (protein + anionic + neutral polysaccharide) systems remain limited. To address this limitation, this study developed and systematically compared binary (WPI + κ-CA) and ternary (WPI + κ-CA + CG) HIPPE systems. The distinct roles of κ-CA and CG in regulating interfacial wettability, microstructure, stability, and rheological properties were elucidated, and the relationships between polysaccharide concentration and 3D printing performance were established. These findings provide a theoretical basis and material-design framework for developing high-performance, customizable food 3D-printing inks.

## 2. Materials and Methods

### 2.1. Materials

Food-grade whey protein isolate (WPI, ≥90% protein) (Nanjing Ximeinuo Biotechnology Co., Ltd., Nanjing, China). Food-grade κ-carrageenan (κ-CA, ≥98%) (Azeres International Trade Co., Ltd., Shanghai, China). Curdlan gum (CG, ≥99%) (Fufeng Group Co., Ltd., Qingdao, China). Analytical grade n-dodecane (Tianjin Kemio Chemical Reagent Co., Ltd., Tianjin, China). Potassium bromide (Shanghai McLean Biochemical Materials Technology Co., Ltd., Shanghai, China). Nile red (≥95%) (Shanghai Yuanye Biotechnology Co., Ltd., Shanghai, China). Nile blue (1% in ethanol) (Shanghai Yuanye Biotechnology Co., Ltd., Shanghai, China).

### 2.2. Preparation of Composite Colloidal Particles

Citrate buffer (20 mM, pH 4.71) was prepared by adjusting the citric acid-to-trisodium citrate ratio. WPI and κ-CA were separately dissolved in citrate buffer, stirred for 2 h, and hydrated overnight at 4 °C to obtain a 4.5% (*w*/*v*) protein suspension and κ-CA suspensions containing 0.9, 1.2, 1.5, 1.8, or 2.1% (*w*/*v*) κ-CA. The WPI and κ-CA solutions were mixed at a 1:2 (*v*/*v*) ratio in centrifuge tubes, vortexed for 3 min, and equilibrated at room temperature for 1 h to form binary self-assembled particles. The final concentrations in the binary system were 1.5% (*w*/*v*) WPI and 0.6%, 0.8%, 1.0%, 1.2%, or 1.4% (*w*/*v*) κ-CA.

For the ternary system, κ-CA and CG powders were premixed and dissolved in the same citrate buffer. After 2 h stirring and overnight hydration at 4 °C, suspensions containing κ-CA (0.9–2.1%, *w*/*v*) and CG (1.2, 1.8, 2.4, 3.0, or 3.6%, *w*/*v*) were obtained. WPI was then mixed with the κ-CA + CG suspension at a 1:2 (*v*/*v*) ratio, vortexed for 3 min, and equilibrated at room temperature for 1 h to form ternary self-assembled particles. The final concentrations in the ternary system were 1.5% (*w*/*v*) WPI, 1.2% (*w*/*v*) κ-CA, and 0.8%, 1.2%, 1.6%, 2.0%, or 2.4% (*w*/*v*) CG. All composite colloidal particles were freeze-dried for 48 h before characterization.

### 2.3. Three-Phase Contact Angle

The three-phase contact angle (θ) of the composite particles was measured using a surface tensiometer (DCAT21, DataPhysics, Munich, Germany), according to Li et al. [14], with slight modifications. Freeze-dried particles were compressed into 2 mm thick tablets and immersed in n-dodecane for 5 min to achieve complete saturation with the organic phase. A deionized water droplet (~2 μL) was deposited onto the tablet surface, and droplet profiles were recorded with a high-speed camera after 30 s equilibration. Contact angles were determined by fitting the droplet contour to the Laplace–Young equation.

### 2.4. Structural Characterization

The structural properties of the freeze-dried particles were characterized by Fourier transform infrared (FTIR) spectroscopy and X-ray diffraction (XRD).

FTIR spectroscopy was performed according to Zhou et al. [15] with slight modifications. Approximately 10 mg sample was mixed with 1000 mg potassium bromide (KBr), ground in an agate mortar, and compressed into transparent pellets. Spectra were collected using an FTIR spectrometer (TENSOR 27, Bruker, Ettlingen, Germany) over 400–4000 cm^−1^ at 4 cm^−1^ resolution with 16 scans per spectrum.

XRD analysis followed Zhou et al. [16] with modifications. Powdered samples were loaded into a sample holder and analyzed using a Bruker D8 Advance A25 X-ray diffractometer (Bruker, Germany). Diffraction patterns were recorded over a 2θ range of 5–60° at 2°/min.

### 2.5. Preparation of Pickering Emulsion

Self-assembled particle suspensions (12 mL) were transferred to a beaker, and n-dodecane or corn germ oil was added to obtain an internal phase volume fraction of 75%. The mixture was sheared using an IKA T25 digital Ultra-Turrax (IKA, Staufen im Breisgau, Germany) at 13,200 rpm for 1 min per cycle, with 30 s intervals between consecutive cycles. The resulting Pickering emulsions were stored in centrifuge tubes and aged at 4 °C for 24 h before analysis.

### 2.6. Droplet Size and Distribution of Pickering Emulsion

Droplet size and distribution were analyzed by optical microscopy and confocal laser scanning microscopy (CLSM). For optical microscopy, Pickering emulsions was placed on a glass slide, covered with a coverslip, and observed using an inverted microscope (Axio Vert.A1, Zeiss, Oberkochen, Germany) equipped with a ×63 objective. Three random fields were captured for each sample, and droplet sizes were measured using Nano Measurer software (version 1.2).

For CLSM, the oil and aqueous phases were stained with Nile red (0.1% *w*/*v*) and Nile blue (0.1% *w*/*v*), respectively. The stained emulsion was placed on a concave slide, covered with a coverslip, and examined using a CLSM (LSM 780, Zeiss, Oberkochen, Germany) equipped with a ×63 objective. Nile red and Nile blue were excited at 514 and 633 nm, respectively. Images were processed using ZEN 2 Lite software.

### 2.7. Stability of Pickering Emulsion

Thermal stability: Pickering emulsions (5 mL) were heated in a 90 °C water bath for 30 min. Samples were collected every 10 min, immediately cooled in an ice bath to room temperature, and observed by optical microscopy to monitor microstructural changes.

Centrifugal stability: Following Li et al. [14] with modifications, Pickering emulsions was centrifuged at 8000 rpm for 10 min at 25 °C using a centrifuge (5810R, Eppendorf, Hamburg, Germany). Phase separation was photographed immediately after centrifugation to evaluate stability.

### 2.8. Moisture Distribution of Pickering Emulsion

Water distribution in Pickering emulsions was analyzed using a low-field nuclear magnetic resonance (NMR) imaging analyzer (NMI20-040V-I, Niumag, Suzhou, China) following Li et al. [17] with minor modifications. Aged Pickering emulsions were equilibrated at room temperature for 1 h, and 3.0 g of each sample was transferred into a 20 mm NMR tube. Transverse relaxation time (T_2_) measurements were performed under the following conditions: 32 °C, SF 20 MHz, TW 6500 ms, SW 100 kHz, NS 8, NECH 12,000, P1 6.52 μs, and P2 13.52 μs. Data were acquired using Niumag software Ver 4.0, and inversion was performed with 10^5^ iterations.

### 2.9. Rheological Characteristics

Following the method of Li et al. [14] with slight modifications, rheological properties were measured using a HAAKE RS600 rheometer (Thermo Fisher Scientific, Karlsruhe, Germany) equipped with a P35 TiL plate (35 mm diameter) and a 1000 μm gap. The temperature was maintained at 25 °C, and silicone oil was applied around the plate to prevent water evaporation.

Flow sweep test: The apparent viscosity of the Pickering emulsion was measured over a shear rate range of 0.1–100 s^−1^.

Stress sweep test: The storage modulus (G′) and loss modulus (G″) of the Pickering emulsion were measured at 1.0 Hz under oscillatory stress ranging from 1 to 1000 Pa. Yield stress was defined as the intersection point where G′ = G″.

Frequency sweep test: The G′ and G″ of the Pickering emulsion were measured at a strain stress of 1 Pa over a frequency range of 0.1–100 Hz.

Three-interval thixotropic test (3ITT): A three-step shear test was conducted at fixed shear rates of 1, 100, and 1 s^−1^ for 120 s each. The recovery ratio was calculated as the ratio of the average steady-state viscosity during the third time interval to that during the first interval under identical time conditions.

### 2.10. Texture Properties

Textural properties were analyzed using a texture analyzer (TA.XT Plus, Stable Micro Systems, Godalming, UK) equipped with a P/36R cylindrical probe (36 mm diameter). Following Feng et al. [18] with slight modifications, 30 mL of Pickering emulsion was transferred to a weighing bottle and equilibrated at room temperature for 1 h. A double-compression cycle was performed at 50% strain with pre-test, test, and post-test speeds of 5.0 mm/s, 0.5 mm/s, and 5.0 mm/s, respectively. Hardness, springiness, cohesiveness, gumminess, and chewiness were calculated from the force–time curves.

### 2.11. 3D Printing

Following Li et al. [14] with slight modifications, 3D printing performance was evaluated using a food-grade 3D printer (FOODBOT D1, Shiyin Technology, Hangzhou, China). A tortoise-shaped model (x: 1.9 cm, y: 3.7 cm, z: 2.8 cm) from the printer’s built-in library was used to evaluate printing accuracy and self-supporting capability. Printing parameters were set as follows: nozzle diameter, 0.8 mm; printing speed, 60% of maximum; filling density, 10%; linear infill pattern; and printing temperature, 25 °C. Printed samples were photographed immediately after printing to assess shape fidelity.

### 2.12. Statistical Analysis

All experiments were performed in at least triplicate. Data are expressed as mean ± standard deviation (SD). Statistical significance was determined by one-way analysis of variance (ANOVA) followed by Tukey’s post hoc test using SPSS 29.0 software (IBM, New York, NY, USA). Differences were considered significant at *p* < 0.05. Graphs were generated using Origin 2024 (OriginLab, Northampton, MA, USA).

## 3. Results and Discussion

### 3.1. Three-Phase Contact Angle

**Figure 1:**
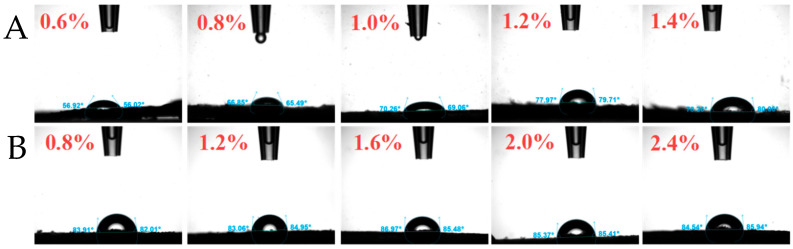
Three-phase contact angles of composite colloidal particles: (**A**) Co-assembled particles of WPI + ĸ-CA; (**B**) Co-assembled particles of WPI + ĸ-CA + CG.

The three-phase contact angle (θ) of composite colloidal particles critically governs interfacial adsorption and emulsion stabilization. As θ approaches 90°, particle desorption energy at the oil–water interface is maximized, resulting in optimal emulsion stability [19]. In the binary system (WPI + κ-CA), increasing κ-CA concentration from 0.6% to 1.2% raised the contact angle from 69.42 ± 0.91° to 78.84 ± 0.87°, after which it plateaued (Figure 1A). This increase suggests that κ-CA interacts with WPI through hydrogen bonding and electrostatic interactions, exposing hydrophobic regions of WPI and enhancing the oil-phase wettability of the composite particles [10]. In the ternary system (WPI + κ-CA + CG), the contact angle increased with CG concentration up to 1.6%, reaching 86.22 ± 0.74°, significantly higher than that of the optimized binary system (*p* < 0.05) (Figure 1B). This enhancement likely results from CG adsorption onto WPI–κ-CA complexes through hydrogen bonding and hydrophobic interactions, further expanding hydrophobic domains and increasing oil-phase affinity. The higher contact angle in the ternary system suggests stronger interfacial anchoring, which likely improves droplet refinement and emulsion stability.

### 3.2. Structural Characterization

**Figure 2:**
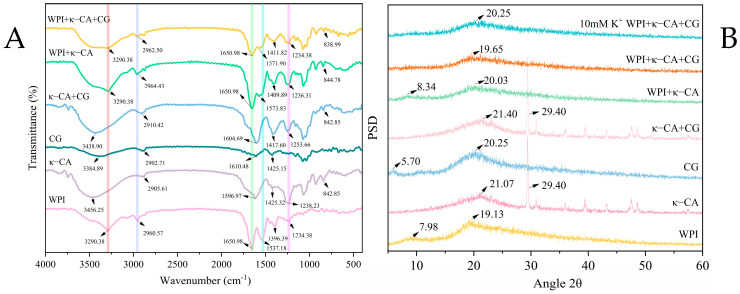
Structural characterization of composite colloidal particles. (**A**) Fourier-transform infrared spectra of composite colloidal particles; (**B**) X-ray diffraction patterns of composite colloidal particles.

To clarify the molecular interactions underlying the altered interfacial wettability, FTIR and XRD analyses were performed on freeze-dried composite colloidal particles (Figure 2). FTIR spectra of the composite particles showed no new absorption peaks relative to the individual components, indicating the absence of covalent cross-linking (Figure 2A). After WPI and κ-CA complexation, shifts appeared at 2960 cm^−1^ (C–H stretching), 1540 cm^−1^ (amide II), and 1240 cm^−1^ (S=O stretching), accompanied by broadening at 3300 cm^−1^ (O–H and N–H stretching). These changes indicate non-covalent interactions, including electrostatic interactions, hydrogen bonding, and hydrophobic associations, between WPI and κ-CA. After CG addition, further shifts occurred at 2960, 1540, 1400, and 842 cm^−1^. Notably, broadening of the 3300 cm^−1^ peak became more pronounced, whereas the 2960 and 1540 cm^−1^ peaks exhibited blue shifts toward higher wave numbers. These results suggest that CG strengthens hydrogen bonding while partially weakening electrostatic interactions between WPI and κ-CA, consistent with the neutral nature of CG [20,21].

XRD analysis showed that all composite colloidal particles exhibited a broad amorphous peak centered at 2θ = 20°, characteristic of disordered molecular arrangements (Figure 2B). The binary composite (WPI + κ-CA) displayed an additional broad peak at 8.34°, which disappeared in the ternary composite, suggesting that CG disrupts both the ordered secondary structure of WPI and the double-helix structure of κ-CA. The ternary composite exhibited only a single broad peak at 19.65°, indicating a fully amorphous structure with enhanced molecular flexibility. This amorphous state may facilitate interfacial unfolding and reduce surface tension during adsorption. The non-covalent interactions and enhanced molecular flexibility observed in the ternary system provide a structural basis for improved interfacial activity and emulsion stability [16,22].

### 3.3. Droplet Size and Distribution of Pickering Emulsion

**Figure 3:**
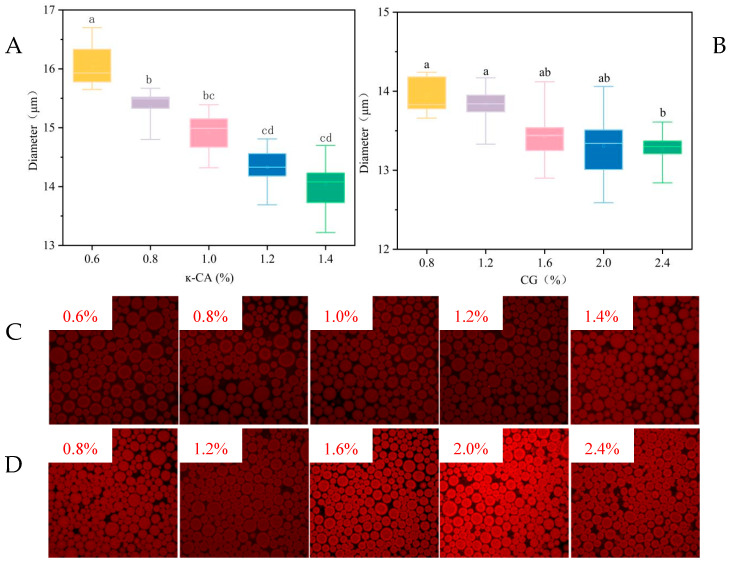
Droplet size distribution and confocal laser scanning microscope (CLSM) images of Pickering emulsions (oil phase shown in red). (**A**,**C**) Pickering emulsions of the WPI + ĸ-CA binary system; (**B**,**D**) Pickering emulsions of the WPI + ĸ-CA + CG ternary system. Different lowercase letters indicate significant differences between samples (*p* < 0.05). The CLSM images were acquired and captured under a 63× objective lens.

Droplet size and uniformity directly reflect emulsification efficiency and are critical determinants of emulsion stability [23]. In the binary system, increasing κ-CA concentration from 0.6% to 1.2% reduced droplet diameter from 16.03 ± 0.30 μm to 14.26 ± 0.28 μm (*p* < 0.05), after which no significant change occurred (Figure 3A). CLSM images confirmed that all emulsions were O/W type, with n-dodecane stained red (Figure 3C). This trend positively correlated with the increased contact angle observed in Section 3.1, suggesting that improved interfacial wettability at 1.2% κ-CA promotes more efficient droplet breakup and stabilization. At lower κ-CA concentrations, insufficient interfacial coverage promotes droplet coalescence, whereas excess κ-CA may saturate the interface without further reducing droplet size [9]. In the ternary system, droplet size decreased significantly only at CG concentrations ≥ 1.6%, reaching 13.52 ± 0.15 μm (*p* < 0.05 vs. the binary system containing 1.2% κ-CA) (Figure 3B). CLSM images further showed greater droplet uniformity than in the binary system (Figure 3D). This synergistic effect likely results from the combined interfacial actions of κ-CA and CG: κ-CA enhances electrostatic stabilization, whereas CG, which possesses intrinsic emulsifying capacity, co-adsorbs at the interface and increases interfacial coverage [13]. The absence of further droplet-size reduction above 1.6% CG may result from increased viscosity limiting particle diffusion to the interface. Consequently, the ternary system is expected to exhibit greater resistance to coalescence, as examined in the following section.

### 3.4. Stability of Pickering Emulsion

**Figure 4:**
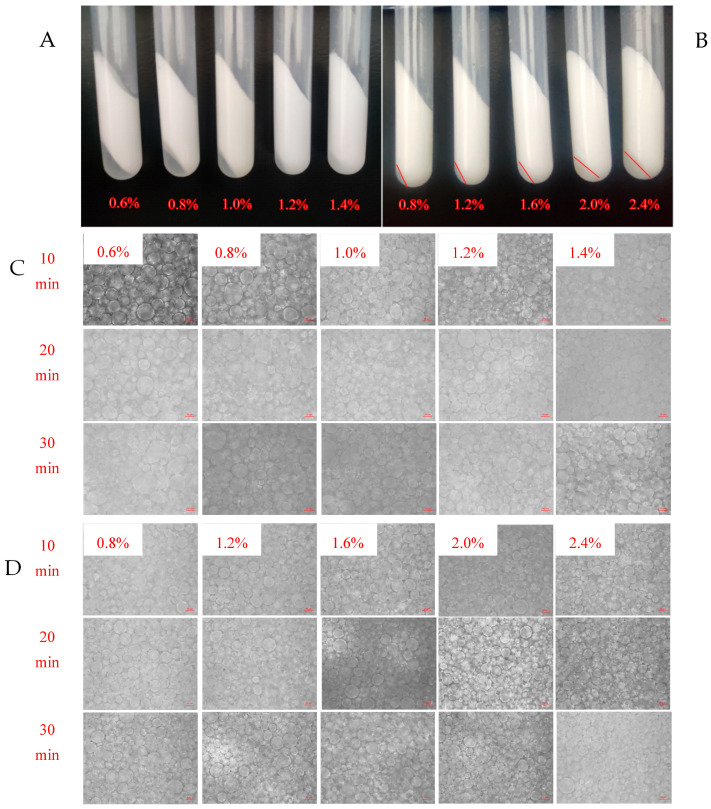
Apparent morphology of Pickering emulsions after centrifugation and microscopic morphology of emulsion droplets under a light microscope (scale bar = 20 μm) (**A**,**C**) Pickering emulsions of the WPI + ĸ-CA binary system; (**B**,**D**) Pickering emulsions of the WPI + ĸ-CA + CG ternary system.

Centrifugal stability reflects emulsion resistance to mechanical stress during storage and transportation [24]. After centrifugation, all emulsions exhibited serum separation (aqueous phase release) without oil separation, confirming their O/W nature (Figure 4A,B). In the binary system, the separated serum layer volume decreased with increasing κ-CA concentration up to 1.2%, which aligned with the decreasing trend of droplet size (Figure 4A). These results demonstrate that at κ-CA concentrations exceeding 1.2%, increased viscosity in the continuous phase and the polysaccharide network structure effectively inhibit droplet coalescence under centrifugal force [9]. No further improvement in stability was observed when the κ-CA concentration exceeded 1.2%, suggesting saturation of the interfacial and continuous-phase networks. In the ternary system, low CG concentrations further reduced serum separation and improved centrifugal stability relative to the optimized binary system. However, serum separation increased when the CG concentration exceeded 2.0% (Figure 4B). FTIR and contact angle analyses showed that CG enhanced particle wettability via hydrogen bonding and hydrophobic interactions, thereby improving interfacial stabilization at appropriate concentrations. Nevertheless, excessive CG likely introduced steric hindrance and disrupted the interpenetrating polysaccharide network in the continuous phase, reducing its ability to restrict droplet migration and increasing phase separation. This trend was consistent with the rheological results presented in Section 3.6.

Thermal stability is critical for food applications involving cooking or pasteurization [24]. In the binary system, heating at 90 °C for 20 min caused minimal microstructural changes (Figure 4C). This improvement can be attributed to the enhanced electrostatic repulsion between droplets provided by κ-CA, which suppresses the heat-induced hydrophobic aggregation of WPI. However, pronounced droplet coalescence and deformation occurred after 30 min of heating, indicating that the binary system cannot withstand prolonged thermal treatment. This heat-induced droplet coalescence aligns with previous reports on protein-polysaccharide stabilized emulsions [24].

In contrast, the ternary system showed no droplet deformation or coalescence after 30 min of heating, regardless of CG concentration (Figure 4D). This enhanced thermal stability stems from the thermal gelation property of CG, which compensates for the decreased viscosity of κ-CA at high temperatures and restricts droplet mobility; additionally, CG suppresses the thermal denaturation-induced aggregation of WPI. The neutral polysaccharide CG may compete with WPI for interfacial adsorption, forming a mixed film that reduces the thermal sensitivity of WPI. Additionally, the CG/κ-CA network in the aqueous phase restricts oil droplet mobility during heating [25].

### 3.5. Moisture Distribution of Pickering Emulsion

**Figure 5:**
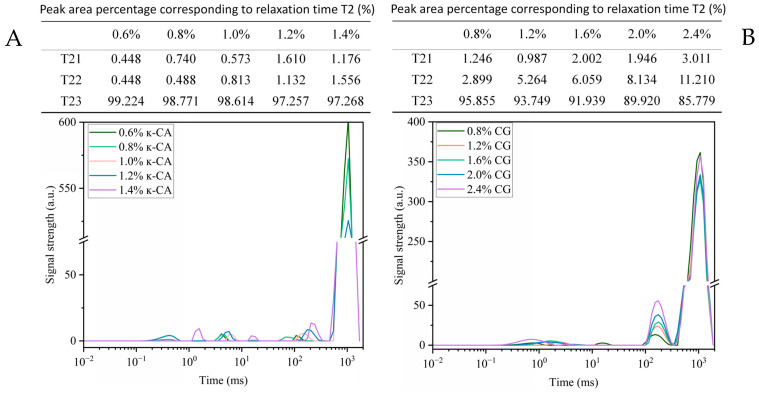
Water distribution in Pickering emulsions determined by low-field NMR. (**A**) Pickering emulsions of the WPI + ĸ-CA binary system; (**B**) Pickering emulsions of the WPI + ĸ-CA + CG ternary system.

Water distribution within the emulsion gel network influences both stability and 3D-printing performance. Low-field NMR identified three water populations: T_21_ (bound water), T_22_ (immobilized water), and T_23_ (free water). A higher T_22_ proportion correlates with improved structural integrity, whereas excess T_23_ promotes interlayer collapse during printing [26]. In the binary system, increasing κ-CA concentration to 1.2% increased T_22_ and correspondingly decreased T_23_ (Figure 5A). This trend agrees with the improved centrifugal stability described in Section 3.4, indicating that κ-CA enhances water immobilization through aqueous-phase network development. However, the limited water-binding capacity of κ-CA alone maintained a relatively high free-water fraction compared with the ternary system. Liu, et al. [27] reported similar findings. In the ternary system, CG addition significantly increased T_22_ and decreased T_23_ relative to the binary system (Figure 5B). This improvement likely arises from two factors: (1) CG forms hydrogen-bonded networks with κ-CA in the aqueous phase, physically entrapping water molecules, and (2) CG possesses high water-absorption capacity, further increasing immobilized water content. These results are consistent with the enhanced thermal stability described in Section 3.4. The ternary system exhibited excellent water-holding capacity, with the thermoreversible gel formed by the two polysaccharides showing considerable potential for improving the rheological and textural properties required for 3D printing.

### 3.6. Rheological Characteristics

Rheological properties simulate the flow and deformation behavior of Pickering emulsions under external forces during extrusion-based 3D printing. Therefore, they are commonly used to evaluate 3D-printing reproducibility [14,28].

**Figure 6:**
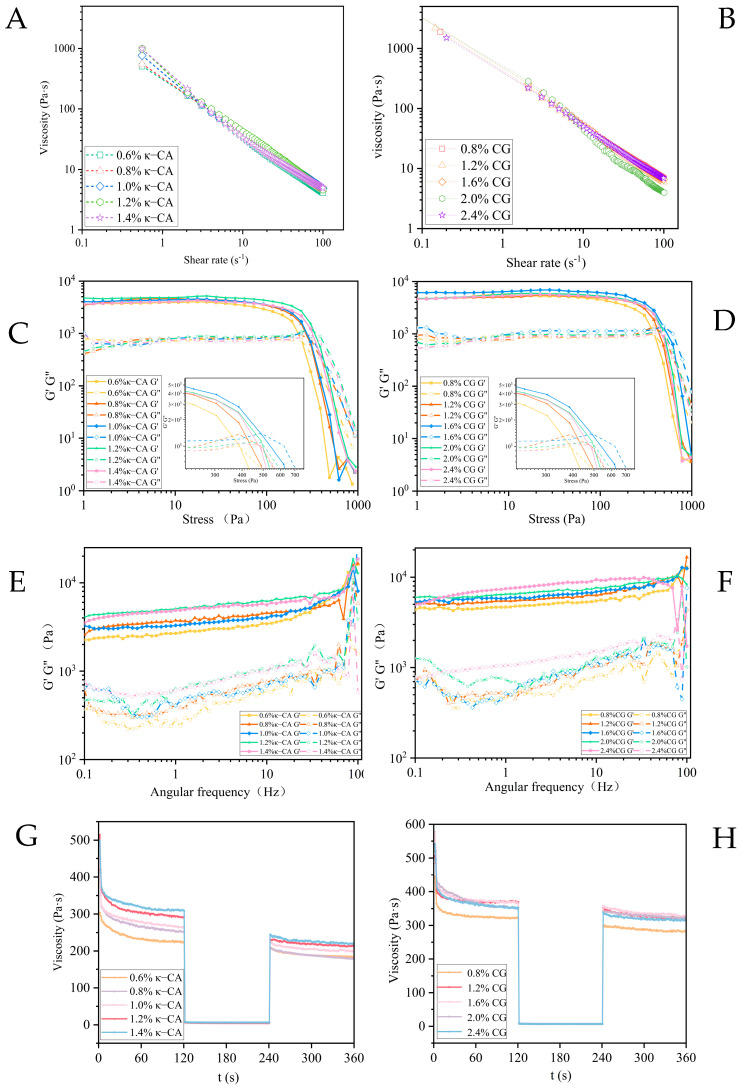
Rheological properties of Pickering emulsions: shear rate–viscosity curves, stress sweep curves, frequency sweep curves, and thixotropic recovery curves. (**A**,**C**,**E**,**G**) Pickering emulsions of the WPI + ĸ-CA binary system; (**B**,**D**,**F**,**H**) Pickering emulsions of the WPI + ĸ-CA + CG ternary system.

Both binary and ternary Pickering emulsions exhibited pronounced shear-thinning behavior (Figure 6A,B), with apparent viscosity decreasing as shear rate increased from 0.1 to 100 s^−1^. This behavior is characteristic of physically crosslinked gel networks that disassemble under shear and recover after shear removal. Such shear-thinning behavior enables smooth emulsion extrusion during printing [29].

Yield stress reflects resistance to deformation under static conditions and is critical for post-printing shape retention [30]. In the binary system, yield stress (defined as the crossover point where G′ = G″) increased with κ-CA concentration up to 1.2%, then plateaued (Figure 6C). The storage modulus (G′) and loss modulus (G″) showed similar trends, whereas tanδ remained ~0.15 across formulations, indicating solid-like elastic behavior (Figure 6E). These results suggest that κ-CA structurally strengthens the emulsion gel by forming an aqueous-phase network. However, the plateau at higher concentrations indicates network saturation beyond a critical κ-CA level. In the ternary system, yield stress, G′, and G″ were significantly higher than in the binary system at optimal concentrations (Figure 6D,F). This enhancement likely results from a synergistic network formed by CG and κ-CA in the aqueous phase. As a long-chain neutral polysaccharide, CG interacts with κ-CA through hydrogen bonding to form an interpenetrating network that reinforces the gel structure. Meanwhile, tanδ remained ~0.14, confirming that the ternary system retained the solid-like properties required for shape fidelity [11,31]. However, at excessively high CG concentrations, G’ and G” increased sharply with shear frequency, indicating that CG weakened the three-dimensional network through steric hindrance (Figure 6F).

Thixotropic recovery reflects the ability of an emulsion to rebuild its network after extrusion and is critical for printing accuracy [31]. In the binary system, increasing κ-CA concentration raised viscosity but reduced the thixotropic recovery ratio (Figure 6G). This trade-off likely arises from the rigid κ-CA double-helix network. At high concentrations, densely entangled κ-CA chains restrict molecular mobility and slow network reformation after shear disruption. In contrast, the ternary system maintained a high recovery ratio even at elevated CG concentrations (Figure 6H). The addition of CG increased network flexibility through hydrogen-bond interactions, enabling faster structural recovery after shear cessation. These results indicate greater molecular flexibility in the ternary system. The combination of high viscosity and rapid recovery makes the ternary system particularly suitable for 3D printing applications [9,11].

### 3.7. Texture Properties

Texture properties, including hardness, springiness, cohesiveness, gumminess, and chewiness, collectively describe the mechanical performance of emulsion gels. Among these, hardness is particularly important for self-support after printing [32].

**Figure 7:**
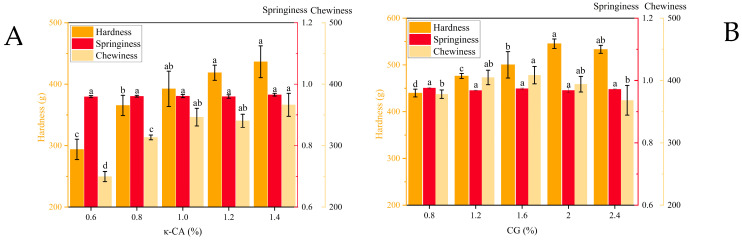
Textural properties of Pickering emulsions. (**A**) Pickering emulsions of the WPI + ĸ-CA binary system; (**B**) Pickering emulsions of the WPI + ĸ-CA + CG ternary system. Different lowercase letters indicate significant differences between samples (*p* < 0.05).

In the binary system, hardness and chewiness increased with κ-CA concentration up to 1.2%, whereas springiness showed no significant change (Figure 7A). This pattern suggests that the emulsion network is dominated by physical crosslinking rather than covalent bonding, consistent with the FTIR results (Section 3.2). The increased hardness also correlated with the higher yield stress observed in Section 3.6, confirming that κ-CA strengthens the gel structure through interfacial and aqueous-phase network formation. In the ternary system, hardness increased significantly with CG concentration up to 1.6%, whereas chewiness initially increased and then declined at higher CG concentrations (Figure 7B). At comparable κ-CA levels, the ternary system exhibited markedly greater hardness than the binary system, reflecting reinforcement by the CG–κ-CA interpenetrating network. However, excessive CG may disrupt κ-CA dispersion, weaken the gel network, and promote droplet sliding, explaining the reduced chewiness at CG concentrations > 1.6%. Similar results were reported by Liu, et al. [27] in pea protein–carrageenan stabilized Pickering emulsions (Figure 7B). The enhanced textural properties of the ternary system, particularly hardness, suggest superior self-supporting capability during 3D printing, as examined below.

### 3.8. 3D Printing

To evaluate the practical applicability of the developed Pickering emulsions, 3D printing performance was assessed using a tortoise-shaped model, with emphasis on printing accuracy (shape fidelity) and structural support (self-standing capability) [33].

**Figure 8:**
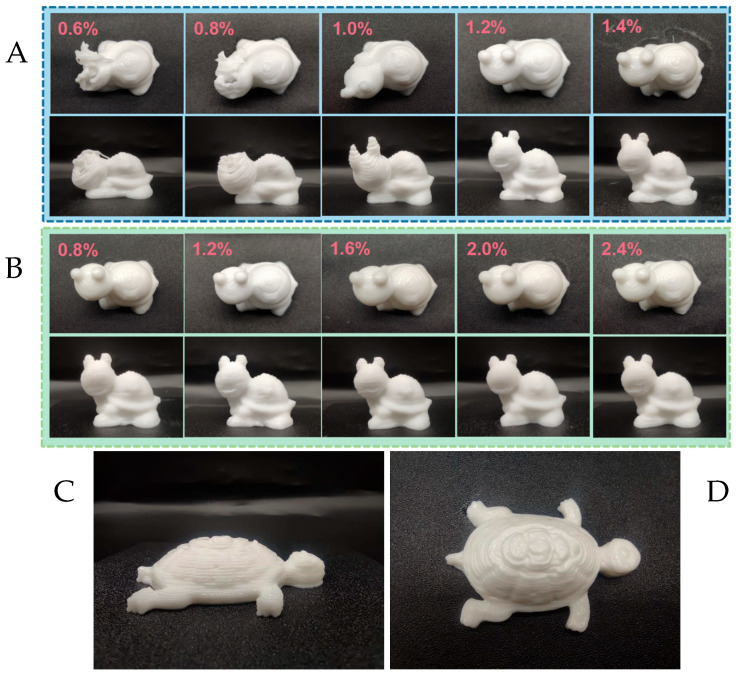
Apparent morphology of 3D-printed Pickering emulsions. (**A**) Pickering emulsions of the WPI + ĸ-CA binary system; (**B**) Pickering emulsions of the WPI + ĸ-CA + CG ternary system. 3D-printed Pickering emulsions prepared using corn germ oil as the oil phase: (**C**) Front view; (**D**) Top view.

In the binary system, printing accuracy improved as κ-CA concentration increased to 1.2% (Figure 8A). At κ-CA concentrations ≤ 0.8%, the printed structures collapsed immediately after extrusion because the yield stress and gel strength were insufficient to support the deposited layers. At the optimal κ-CA concentration of 1.2%, the printed tortoise retained its overall shape, although slight edge blurring and line distortion were still observed. This optimal performance corresponded to the peak contact angle (Section 3.1), minimum droplet size (Section 3.3), and maximum yield stress (Section 3.6) observed at 1.2% κ-CA, indicating that printing accuracy depends on the combined effects of interfacial coverage and gel strength. Similar findings were reported by Bi, et al. [31]. The ternary WPI–κ-CA–CG system exhibited significantly greater shape fidelity and structural integrity than the binary system, with the optimal performance achieved at 1.6% CG (Figure 8B). The printed tortoise exhibited excellent shape fidelity with minimal distortion, surpassing the binary system in both accuracy and structural support. Notably, the optimal CG concentration (1.6%) coincided with the maximum contact angle (Section 3.1), minimum droplet size (Section 3.3), and peak hardness (Section 3.7), further supporting the structure–property relationships established in this study. Yu, et al. [11] similarly reported that the neutral polysaccharide konjac gum provided better 3D printing accuracy than xanthan gum in SPI emulsion systems.

To further evaluate its feasibility for food applications, the optimized ternary HIPPE was prepared using food-grade corn germ oil as the oil phase and subjected to 3D printing (Figure 8C,D). These results confirm that the optimized ternary formulation is compatible with edible oils and suitable for food 3D printing applications. The performance achieved here matches or exceeds that of previously reported protein–polysaccharide-stabilized HIPPEs systems [5,14], verifying the effectiveness of the proposed dual-polysaccharide reinforcement strategy.

Overall, the 3D printing results demonstrate that κ-CA and CG play complementary roles in regulating emulsion printability: κ-CA primarily increases yield stress and gel hardness, thereby enhancing the self-supporting capacity of printed structures; whereas CG mainly improves thixotropic recovery rate and network flexibility, resulting in higher printing accuracy and shape fidelity. The combination of the two polysaccharides achieves a balanced improvement in structural rigidity and shear recovery, resulting in superior 3D printing performance.

## 4. Conclusions

This study systematically constructed and compared binary (WPI + κ-CA) and ternary (WPI + κ-CA + CG) HIPPEs systems with tunable 3D printing performance. The results show that κ-CA and CG exert distinct yet complementary effects on emulsion properties: κ-CA enhances interfacial adsorption and structural rigidity through electrostatic interactions and hydrogen bonding, whereas CG improves interfacial flexibility and shear recovery through hydrogen-bonded network formation. The optimal 3D-printing formulation contained 1.2% κ-CA and 1.6% CG in the ternary system. At these concentrations, the ternary HIPPEs exhibited a three-phase contact angle of 86.22 ± 0.74°, a droplet size of 13.52 ± 0.15 μm, and greater thermal stability, shear recovery, and hardness than the binary system. This enhanced printability was associated with the synergistic formation of an interpenetrating polysaccharide network in the aqueous phase, which reinforced gel structure while preserving extrusion flexibility. Mechanistically, the results indicate that anionic polysaccharides (κ-CA) and neutral polysaccharides (CG) regulate HIPPEs printability through distinct pathways: κ-CA controls yield stress and self-supporting capacity, whereas CG governs thixotropic recovery and printing accuracy. These findings provide a mechanistic basis for designing polysaccharide-reinforced emulsion inks with tailored rheological and printing properties.

Despite these advances, several limitations require further investigation. First, the microstructural organization of ternary particles at the oil–water interface has not been directly visualized; techniques such as cryo-electron microscopy (Cryo-EM) and atomic force microscopy (AFM) could verify the proposed co-assembly mechanism. Second, the long-term storage and thermal stability of 3D-printed products under commercial processing conditions remain unclear. Future studies should also assess production scalability and explore applications of this HIPPEs system in functional foods, including texturized dysphagia diets and bioactive-compound delivery systems.

## Data Availability

The original contributions presented in the study are included in the article, further inquiries can be directed to the corresponding author.
